# Effect of β-mannanase supplementation in low-energy diets containing palm kernel meal on productive performance, egg quality, intestinal morphology, and liver characteristics in laying hens raised under heat stress conditions

**DOI:** 10.5713/ab.25.0561

**Published:** 2025-10-22

**Authors:** Charline Mugeniwayesu, Ju Hye Kim, Kang Hyeon Kim, Eun Cheol Lee, Dong Yong Kil

**Affiliations:** 1 Department of Animal Science and Technology, Chung-Ang University, Anseong, Korea

**Keywords:** β-mannanase, Heat Stress, Laying Hen, Low-energy Diet, Palm Kernel Meal, Productive Performance

## Abstract

**Objective:**

This study aimed to investigate the effect of β-mannanase (MN) supplementation in low-energy diets containing palm kernel meal (PKM) on productive performance, egg quality, intestinal morphology, and liver characteristics in laying hens under heat stress (HS) conditions.

**Methods:**

Four hundred 37-wk-old Hy-Line Brown laying hens were randomly allotted to 5 dietary treatments with 8 replicates for 8 wks of the feeding trial conducted under a cyclic HS condition. The positive control (PC) diet was prepared, whereas the low-energy negative control (NC) diet was formulated to contain decreased AME_n_ by 100 kcal/kg than PC diets. An additional low-energy diet was prepared by including 5.0% PKM, and it was supplemented with either 0.05% or 0.10% MN.

**Results:**

Feeding low-energy diets containing PKM increased (p<0.05) feed conversion ratio (FCR), but MN supplementation did not affect FCR in laying hens under HS conditions. Egg yolk color was improved (p<0.05) by feeding low-energy diets containing PKM, regardless of MN supplementation with no differences in other egg quality among treatments. The supplementation of 0.10% MN in PKM-containing low-energy diets showed lower (p<0.05) blood heterophil to lymphocyte ratio (H:L) ratio than NC diet, but exert blood H:L ratio comparable to PC diet. A linear trend for increased villus height (VH) was observed (p<0.05) by increasing MN supplementation in PKM-containing low-energy diets with the greatest VH was found in 0.10% MN supplementation.

**Conclusion:**

Feeding low-energy diets containing 5.0% PKM decreased feed efficiency in laying hens under HS conditions. However, MN supplementation in PKM-containing low-energy diets had no beneficial effects on laying performance. Low-energy diets, irrespective of PKM inclusion, exacerbate stress responses and impair jejunal morphology. However, 0.10% MN supplementation in PKM-containing low-energy diets ameliorated stress responses and improved jejunal morphology in laying hens under HS conditions.

## INTRODUCTION

The animal industry is increasingly challenged by heat stress (HS) as a significant environmental stressor that adversely affects animal production and health. In particular, poultry are highly vulnerable to HS because of their limited development of sweat glands and high feather coverages, which hampers their ability to regulate body temperature [1]. This compromised thermoregulation often results in physiological imbalances, abnormal behaviors, and metabolic dysfunctions, thereby impairing productive performance, product quality, and health in poultry [2,3]. Therefore, considering an anticipated increase in the prevalence and severity of HS due to the recent climate change, the development of efficient strategies to mitigate the negative outcome of HS in poultry is imperative.

In addition to challenges posed by HS, there is a growing demand to reduce feed costs with the aim of achieving economical poultry production, which is often accomplished by lowering energy levels and increasing use of cost-effective alternative ingredients in poultry diets. However, such alternative ingredients frequently contain high amounts of non-starch polysaccharides (NSPs), which are recognized as antinutritional factors that impair nutrient utilization and subsequently productive performance in poultry [4,5]. Furthermore, it is suggested that this undigestible NSPs may elevate heat production in poultry body due to enhancing microbial fermentation in the gastrointestinal tract (GIT) [6,7], potentially exacerbating the negative impact of HS in poultry under HS conditions. Therefore, effective nutritional regimens should be developed to mitigate adverse effects of including high-NSP ingredients in poultry diets, particularly under HS conditions.

Palm kernel meal (PKM) is appreciated an economical ingredient in poultry feeds, offering considerable nutritional value through its high protein and energy contents at the relatively low cost, although some nutrients, such as limiting amino acids, are not well balanced [8,9]. Previous research reported that up to 10% PKM as a replacement of corn and soybean meal can be included in poultry diets without sacrificing poultry productivity [8,9]. However, PKM contains high concentrations of β-mannan, an antinutritional NSP, that adversely affects nutrient digestion and utilization in the GIT of poultry, leading to a limitation of high inclusion levels of PKM in poultry diets [5,10]. As a possible solution to mitigate the negative effects of PKM-containing diets, dietary β-mannanase (MN) supplementation has become a common practice because MN can effectively break down the β-mannan backbone, releasing mannanoligosaccharides (MOS) and mannose subunits in the GIT [11,12]. Several previous studies have indicated that MN supplementation in low-energy diets containing high-mannan ingredients such as PKM and copra meal exerts positive effects on nutrient utilization and productive performance in poultry [4,13,14], although beneficial effects of dietary MN supplementation are still variable [15]. To our knowledge, however, no studies have examined the effect of MN supplementation in PKM-containing diets on productive performance and health in laying hens, particularly under HS conditions.

Therefore, the present study aimed to investigate the effect of MN supplementation in low-energy diets containing PKM on productive performance, egg quality, jejunal morphology, stress responses, and liver characteristics in laying hens raised under HS conditions.

## MATERIALS AND METHODS

### Animals, diets, and experimental design

A total of four hundred 37-wk-old Hy-Line Brown laying hens were assigned to 1 of 5 dietary treatments with 8 replicates per treatment in a completely randomized design. Each replicate consisted of 10 consecutive cages with 1 laying hen being housed per cage measuring 37×30×40 cm (width× length×height). Prior to the commencement of the study, all hens were fed a common layer diet under normal environment conditions. The positive control (PC) diet was prepared primarily with corn and soybean meal to contain the recommended levels of energy (i.e., 2,900 kcal/kg AME_n_) and all nutrients for the economic performance of laying hens, according to the Hy-Line Brown nutritional guideline (Table 1) [16]. The low-energy negative control (NC) diet was formulated mainly with corn and soybean meal to contain 2,800 kcal/kg AME_n_, while ensuring that the concentrations of all nutrients, including digestible essential amino acids, total calcium, and available phosphorus, were equivalent to those in the PC diet. An additional low-energy diet was also prepared by including 5.0% PKM with a partial replacement of corn and soybean meal in the low-energy NC diet. Nutritional compositions of the PKM used in this study were reported in our previous study [17]. This PKM-containing low-energy diet was designed to maintain the same concentrations of energy and nutrients to those in the low-energy NC diet. Finally, dietary MN (CTCZYME; declared activity of 800,000 U/kg; CTCBIO) was supplemented to the PKM-containing low-energy diet at 2 different levels of 0.05% and 0.10% in replace of celite. All diets were prepared in mash form.

All hens were exposed to a cyclic HS condition during 8 wks of the feeding trial. The average room temperature was maintained at 31±0.7°C for 8 h/d and 26±1.7°C for the remaining time. The average relative humidity (RH) was 86± 6.9% during the experiment. The mechanical ventilation and heating devices were used to control the room temperature and RH. The HS index calculated using average room temperature and RH based on the Hy-Line Brown management guideline [16], was approximately 82.0 during the entire experiment, indicating that laying hens raised in this study were exposed to severe HS conditions. The diet and water were provided *ad libitum* and a 16-h lighting schedule (16 L:8 D) was implemented throughout the experiment.

### Productive performance and egg quality

Productive performance, including hen-day egg production, egg weight, egg mass, and broken and shell-less egg production rate was recorded daily. The feed intake (FI) and feed conversion ratio (FCR) were determined at the end of the experiment. Egg quality, including eggshell color, egg yolk color, eggshell strength, eggshell thickness, and Haugh unit, was analyzed using 10 eggs per replicate with 5 eggs per d at the end of experiment. Eggshell color was determined using the eggshell color fan (Samyangsa) with scales from 1 to 15, while egg yolk color, eggshell strength, eggshell thickness, and Haugh unit were measured using a digital egg tester (DET-6000; Nabel). The detailed procedures for measuring performance and egg quality were outlined in our previous study [17].

### Sample collection and analysis

At the conclusion of the experiment, individual body weight (BW) of all hens was measured following an 8-h overnight fasting. One hen per replicate, with its BW approximating the average BW of each replicate, was selected and euthanized by CO_2_ asphyxiation (i.e., exposure to 90% CO_2_ for 2 min) for sample collections of the blood, liver, and jejunum.

Blood samples were immediately obtained by a heart puncture and collected in a 6.0-mL BD vacutainer tube (BD). The heterophil to lymphocyte ratio (H:L ratio) in the blood was determined as an indicator of stress responses, according to the method described by Lentfer et al [18]. This analysis was performed at the Biotechnology (BT) research facility center, Chung-Ang University.

Jejunal morphology was measured by the method of our previous study [5]. Briefly, jejunum samples were flushed and stored in 10% buffered formalin. A 5-mm section of each sample was placed onto a slide glass, stained with hematoxylin and eosin, and examined under a light microscope. Villus height (VH), villus width (VW), crypt depth (CD), and VH to CD ratio (VH:CD) were measured with 20 measurements per replicate.

Liver characteristics were measured using the method of our previous study [9]. The liver was examined to assign a subjective fatty liver score on a scale from 1 to 5 (1 = dark red; 5 = yellowish red). The liver hemorrhagic score was also analyzed using a scale from 0 to 5 (0 = normal liver; 5 = large and extensive hemorrhages). Three observers conducted the liver scoring in a blinded manner. In addition, the Commission Internationale de l’Eclairage (CIE) color scales for the lightness (L*), redness (a*), and yellowness (b*) in the liver were measured using a colorimeter (Model CR-10; Konica Minolta Optics).

### Statistical analysis

All data were analyzed using one-way ANOVA in a completely randomized design by GLM procedure of SAS (SAS Institute). Each replicate served as an experimental unit for all analyses. Outlier data were checked by the UNIVARIATE procedure of SAS. The values presented in tables represented means with pooled standard error of the mean. If a significant treatment effect was identified, treatment means were compared using Duncan’s multiple-range test. Statistical significance was set at p<0.05.

## RESULTS AND DISCUSSION

### Productive performance

Productive performance, including hen-day egg production, egg weight, egg mass, FI, and broken and shell-less egg production rate, in laying hens raised under HS conditions was not affected by dietary treatments (Table 2). However, hens fed low-energy NC diets yielded a similar FCR value to those fed PC diets. Hens fed PKM-containing low-energy diets without MN supplementation had FCR comparable to those fed low-energy NC diets, but had greater (p<0.05) FCR than those fed PC diets. Increasing MN supplementation in PKM-containing low-energy diets did not influence FCR in laying hens under HS conditions; however, 0.10% MN supplementation in PKM-containing low-energy diets resulted in greater (p<0.05) FCR than PC and NC treatments.

The observation of no negative impacts on productive performance in laying hens under HS conditions when energy level was reduced by 100 kcal/kg AME_n_ in corn-soybean meal-based diets may indicate that a reduction of up to 100 kcal/kg AME_n_ may be feasible without compromising productive performance, provided that all essential nutrient concentrations satisfy their recommendation levels. This finding may be attributed to the physiological adaptation of laying hens to low-energy diets under HS conditions possibly by reducing FI to minimize heat production and adjusting nutrient utilization [3], which is consistent with the previous finding by Mckee et al [19]. However, inclusion of 5.0% PKM in low-energy diets increased FCR in laying hens under HS conditions compared with PC diets with recommended energy levels. This result contrasts with our earlier findings that feeding low-energy and low-protein diets formulated with 5.0% high-mannan ingredients, including PKM and copra meal, had no negative impacts on productive performance in laying hens under standard environmental conditions [17]. The reason for the negative impact of PKM in this study is likely due to high amounts of fiber in PKM, which may enhance heat production in the body of laying hens [6,7], thereby exacerbating HS in laying hens raised under HS conditions. Therefore, it may be suggested that inclusion of 5.0% PKM in low-energy diets impairs laying performance if hens were raised under HS conditions.

### Egg quality

Egg quality, including eggshell color, eggshell strength, eggshell thickness, and Haugh unit, in laying hens under the current HS conditions was not influenced by dietary treatments (Table 3). Notably, hens fed low-energy NC diets exhibited a greater (p<0.05) egg yolk color than those fed PC diets. Furthermore, PKM-containing low-energy diets without MN supplementation led to a greater (p<0.05) egg yolk color than low-energy NC diets. Nevertheless, dietary supplementation of 0.05% or 0.10% MN in PKM-containing low-energy diets did not further enhance egg yolk color in laying hens under the current HS conditions.

The HS has been reported to exert adverse impacts on physiological responses and metabolic processes in laying hens, thereby compromising egg quality [3,20]. This adverse effect of HS on egg quality has been involved in decreased FI, respiratory alkalosis, reduced blood flow to the shell gland, and impaired calcium metabolism [21–23]. Nevertheless, most egg quality values measured in this study fell within the typical range for the normal egg quality in laying hens. Therefore, it is suggested that egg quality in laying hens under the current HS conditions may not be adversely affected by reduction in dietary energy, inclusion of 5.0% PKM, or dietary MN supplementation. Interestingly, this study revealed that PKM-containing low-energy diets, irrespective of MN supplementation, resulted in improved egg yolk color compared with PC or low-energy NC diets. This improvement is mainly attributed to differences in ingredient compositions used for treatment diets. The PKM-containing low-energy diets were formulated with high amounts of corn gluten meal and 5.0% PKM, which is known for their high concentrations of carotenoids, a key pigment for egg yolk coloration [24,25]. Consistent with this finding, our previous study also reported that feeding high-mannan diets containing PKM and copra meal increased egg yolk coloration in laying hens [17]. In the current study, however, dietary MN supplementation, regardless of its supplemental levels did not lead to further improvements in egg yolk color in hens fed PKM-containing low-energy diets, indicating that dietary MN supplementation does not influence carotenoid absorption and utilization in laying hens.

### Jejunal morphology

Hens fed PC diets under the current HS conditions had a similar VH to those fed low-energy NC diets or PKM-containing low-energy diets without MN supplementation; however, hens fed PKM-containing low-energy diets with 0.10% MN supplementation had a greater (p<0.05) VH than those fed low-energy NC diets or PKM-containing low-energy diets without MN supplementation (Table 4). Notably, a linear trend was observed, indicating that VH was increased with higher levels of MN supplementation in PKM-containing low-energy diets. Moreover, PKM-containing low-energy diets, regardless of MN supplementation, led to a less (p<0.05) CD than PC diets; however, increasing MN supplementation in PKM-containing low-energy diets did not affect CD. The VW and VH:CD ratios were not affected by dietary treatments. Nonetheless, a numerical improvement in the VH:CD ratio was observed with increasing supplementation of MN in PKM-containing low-energy diets.

The observation for an improvement in VH by MN supplementation in PKM-containing low-energy diets may be involved in the degradation of β-mannan, which is abundant in PKM, into low-viscosity and low-molecular weight MOS in the GIT because MOS has been reported to exert a prebiotic effect on promoting intestinal health and enhancing nutrient absorption by stimulating intestinal epithelial growth in poultry [5,12,26,27]. However, although PKM-containing low-energy diets, regardless of MN supplementation, decreased CD compared with PC diets, yet dietary MN supplementation did not lead to any further reduction in CD. The underlying reason for this observation remains unclear; however, it potentially reflects an adaptive physiological response involving altered mucosal turnover and reduced crypt cell proliferation [28]. Moreover, the absence of differences in CD by dietary MN supplementation may suggest that dietary MN and its end product (i.e., MOS) in the GIT are likely more associated with promoting the villus growth rather than modulating crypt cell proliferation [29,30]. Consequently, the current finding of a numerical increase in VH:CD ratios by dietary MN supplementation in PKM-containing low-energy diets is likely caused by increased VH with little changes in CD. Although improved intestinal morphology has been often linked to improved productive performance in laying hens [31], however, the present study found no beneficial effect of dietary MN supplementation in PKM-containing low-energy diets on laying performance.

### Blood heterophil to lymphocyte ratio

**Figure 1:**
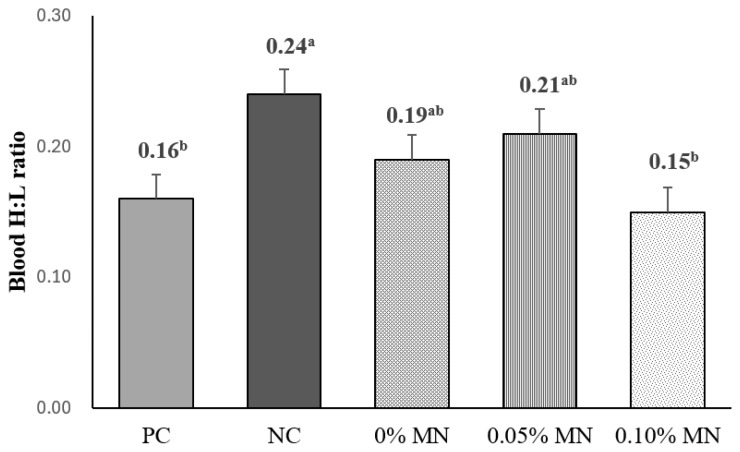
Effect of β-mannanase (MN) supplementation in low-energy diets containing palm kernel meal (PKM) on blood heterophil to lymphocyte (H:L) ratio in laying hens raised under heat stress conditions. Data are least squares means of 8 observations per treatment. PC, recommended-energy diets with 2,900 kcal/kg AME_n_; NC, low-energy diets with 2,800 kcal/kg AME_n_; 0% MN, low-energy diets containing 5% PKM without MN supplementation; 0.05% MN, 0.05% MN supplementation in low-energy diets containing 5% PKM; 0.10% MN, 0.10% MN supplementation in low-energy diets containing 5% PKM. ^a,b^ Means with different superscripts differ (p<0.05).

Hens fed PC diets under the HS conditions had lower (p<0.05) blood H:L ratio than those fed low-energy NC diets (Figure 1). However, PKM-containing low-energy diets without MN supplementation or with 0.05% MN supplementation resulted in a similar blood H:L ratio to low-energy NC diets. Interestingly, 0.10% MN supplementation in PKM-containing low-energy diets showed lower (p<0.05) blood H:L ratio than NC diets, but exert a blood H:L ratio comparable to PC diets.

The blood H:L ratio is widely acknowledged as a stress indicator in poultry, with elevated values reflecting heightened stress responses [1]. Therefore, the current observation for increasing blood H:L ratio by feeding low-energy NC diets than feeding PC diets may indicate that decreasing energy levels by 100 kcal/kg in diets may increase stress responses in laying hens under HS conditions. However, this result contrasts with our previous findings that 100 kcal/kg AME_n_ reduction in diets had no effects on blood H:L ratio in laying hens under standard environmental conditions [17]. This discrepancy may be associated with the fact that HS conditions may increase energy requirements in laying hens, thereby augmenting stress responses when consuming low-energy diets under HS conditions. It was also reported that energy and nutrient deficiencies exacerbate HS responses in broiler chickens [32]. Interestingly, 0.10% MN supplementation in PKM-containing low-energy diets showed a similar blood H:L ratio to PC diets, revealing an indirect evidence that 0.10% MN supplementation in PKM-containing low-energy diets may improve energy utilization in laying hens under HS conditions [7,14]. This hypothesis may also be supported by the current observation that 0.10% MN supplementation in PKM-containing low-energy diets increased VH and VH:CD ratio, reflecting increased absorptive area in the small intestine. Notwithstanding, in this study, these beneficial effects were not associated with improvements in laying performance.

### Liver visual characteristics

The subjective color and hemorrhagic scores in the liver of laying hens under the current HS conditions were not affected by dietary treatments (Table 5). However, a* values in the liver for hens fed PKM-containing low-energy diets with 0.10% MN supplementation were greater (p<0.05) than for those fed PC or low-energy NC diets. However, PKM-containing low-energy diets without MN or with 0.05% MN supplementation displayed similar a* values in the liver to other treatment diets. No differences in L* and b* values were observed among dietary treatments.

The incidence of fatty liver is a significant concern in the layer industry. HS has been identified as a potential contributor to fatty liver development in laying hens [33,34]. A possible solution to mitigate fatty liver incidence in laying hens may be to increase fiber concentrations in layer diets because feeding high-fiber diets has been reported to help regulate lipid transports and mitigate fatty liver progression in poultry [35,36]. Our previous study also indicated that feeding high-fiber diets to laying hens decreased hepatic fat accumulations, demonstrating preventive effects against fatty liver incidence in laying hens [35]. Moreover, decreasing energy levels in layer diets have been often practiced to mitigate fatty liver development in laying hens [37,38].

In the current study, therefore, it was anticipated that feeding low-energy diets containing fibrous PKM exerted a protective effect on fatty liver development in laying hens under HS conditions. However, we failed to find such a beneficial effect in this study based on liver visual characteristics, although a* values in the liver color were numerically increased by feeding low-energy diets containing PKM. Therefore, the lack of positive effects is difficult to explain; however, as suggested by Han et al [38], the type of energy source may be more related to lipid metabolism in laying hens than total energy intake. Moreover, we speculated that the relatively younger hens used in this study may not exhibit obvious fatty liver development at this production stage because fatty liver incidence is more prevalent in older hens [35]. Dietary supplementation of 0.05% MN in PKM-containing low-energy diets had no effects on liver visual characteristics in laying hens under the current HS conditions. Nonetheless, 0.10% MN supplementation improved a* values in the liver compared with PC and NC diets, suggesting the possibility of improving liver health in laying hens under HS conditions.

## CONCLUSION

Feeding low-energy diets containing 5.0% PKM decreased feed efficiency in laying hens under HS conditions, while 0.05% and 0.10% MN supplementation in PKM-containing low-energy diets had no positive effects on productive performance, indicating that inclusion of PKM in diets should be approached with caution for laying hens under HS conditions, regardless of dietary MN supplementation. Low-energy diets, irrespective of PKM inclusion, may exacerbate stress responses and impair jejunal morphology in laying hens under HS conditions. However, 0.10% MN supplementation in PKM-containing low-energy diets ameliorated stress responses and improved jejunal morphology despite little beneficial effects on productive performance in laying hens under HS conditions. Further research may be required to investigate the interactive effect of varying levels of PKM inclusion and dietary MN supplementation across different energy reduction in diets for laying hens raised under HS conditions.

## Figures and Tables

**Table 1:** Composition and nutrient concentrations of experimental diets

Items	PC	NC	PKM
Ingredients (%)			
Corn	60.46	64.05	61.67
Soybean meal, 44.6% CP	20.19	17.61	10.09
Corn gluten meal	3.38	4.67	8.70
Palm kernel meal (PKM)	0.00	0.00	5.00
Soybean oil	3.00	0.51	0.58
Monocalcium phosphate	1.58	1.58	1.59
Limestone	9.21	9.23	9.24
L-Lysine HCl (78.5%)	0.14	0.21	0.37
DL-Methionine (98.5%)	0.21	0.20	0.18
L-Threonine (98.5%)	0.04	0.05	0.08
L-Tryptophan (98.5%)	0.02	0.03	0.06
L-Valine (98.5%)	0.01	0.01	0.03
Celite	0.20	0.20	0.20
Salt	0.20	0.20	0.20
Choline chloride	0.33	0.33	0.33
NaHCO_3_	0.34	0.34	0.22
K_2_CO_3_	0.30	0.40	0.80
Coccidiostat	0.05	0.05	0.05
Antioxidant	0.05	0.05	0.05
Vitamin premix1)	0.15	0.15	0.15
Mineral premix2)	0.15	0.15	0.15
Total	100.00	100.00	100.00
Energy and nutrient contents3)			
AME_n_ (kcal/kg)	2,900	2,800	2,800
CP (%)	16.00	16.00	16.00
Digestible Lys (%)	0.74	0.74	0.74
Digestible Met (%)	0.45	0.45	0.45
Digestible Met+ Cys (%)	0.66	0.66	0.66
Digestible Thr (%)	0.52	0.52	0.52
Digestible Trp (%)	0.16	0.16	0.16
Digestible Arg (%)	0.84	0.80	0.72
Digestible Ile (%)	0.57	0.56	0.53
Digestible Val (%)	0.65	0.65	0.65
Digestible Gly+Ser (%)	1.19	1.17	1.11
Total calcium (%)	3.82	3.82	3.82
Available phosphorus (%)	0.37	0.37	0.37
DEB (mEq/kg)4)	200.00	200.00	200.00
Analyzed nutrient contents5)			
CP (%)	16.40	16.70	17.10
ADF (%)	1.57	1.14	3.10
NDF (%)	9.08	9.22	11.75

PC, corn-soybean meal-based diet with energy (2,900 kcal/kg AME_n_); NC, corn-soybean meal-based diets with low-energy (decreased AME_n_ by 100 kcal/kg than PC diets); PKM, low-energy diets (decreased AME_n_ by 100 kcal/kg than PC diets) formulated with 5% PKM.

1) Provided per kg of the complete diet: vitamin A, 19,500 IU (retinyl acetate); vitamin D_3_, 6,000 IU; vitamin E, 45 mg/kg; vitamin K_3_, 5.0 mg (menadione dimethylpyrimidinol); vitamin B_1_, 5.0 mg; vitamin B_2_, 11.0 mg; vitamin B_6_, 8.0 mg; vitamin B_12_, 30.0 μg; vitamin B_5_, 18 (pantothenic acid); folic acid, 2.0 mg; antioxidant (BHT), 150 μg; biotin, 230 μg; niacin, 75 mg.

2) Provided per kg of the complete diet: iron, 90 mg (FeSO_4_); zinc, 150 mg (ZnSO_4_); manganese, 180 mg (MnO); copper, 24 mg (CuSO_4_); cobalt, 1,500 μg (CoSO_4_); selenium, 450 μg (Na_2_SeO_3_); iodine, 2 mg [Ca (IO_3_)_2_].

3) Calculated values from Centraal Veevoederbureau (CVB) [39].

4) Dietary electrolyte balance.

5) Analyzed nutrient concentrations determined using AOAC methods [40].

**Table 2:** Effect of β-mannanase (MN) supplementation in low-energy diets containing palm kernel meal (PKM) on productive performance in laying hens raised under heat stress conditions

Item	Dietary treatments1)					SEM	p-value

	PC	NC	PKM				

			0% MN	0.05% MN	0.10% MN		
HD (%)	90.8	90.3	89.3	88.6	88.2	1.30	0.588
EW (g)	60.0	60.0	58.8	59.1	58.6	0.47	0.126
EM (g)	54.5	54.2	52.5	52.3	51.7	0.84	0.100
FI (g/hen/d)	104	105	105	104	105	0.6	0.337
FCR (g/g)	1.91^c^	1.94^bc^	2.01^ab^	1.99^ab^	2.03^a^	0.028	0.025
BS (%)	0.23	0.66	0.46	0.42	0.41	0.243	0.812

Data are least squares means of 8 observations per treatment.

1) PC, recommended-energy diets with 2,900 kcal/kg AME_n_; NC, low-energy diets with 2,800 kcal/kg AME_n_; 0% MN, low-energy diets containing 5% PKM without MN supplementation; 0.05% MN, 0.05% MN supplementation in low-energy diets containing 5% PKM; 0.10% MN, 0.10% MN supplementation in low-energy diets containing 5% PKM.

a–c Means with different superscripts within a row differ (p<0.05).

SEM, standard error of the mean; HD, hen-day egg production; EW, egg weight; EM, egg mass; FI, feed intake; FCR, feed conversion ratio; BS, broken and shell-less egg production rate.

**Table 3:** Effect of β-mannanase (MN) supplementation in low-energy diets containing palm kernel meal (PKM) on egg quality in laying hens raised under heat stress conditions

Item		Dietary treatments1)					SEM	p-value

		PC	NC	PKM				

				0% MN	0.05% MN	0.10% MN		
Eggshell color (Shell color fan)		10.5	10.6	10.4	10.4	10.3	0.29	0.986
Eggshell color (CIE Lab value)	L*	50.6	50.6	50.8	50.2	50.5	0.54	0.937
	a*	20.9	21.0	20.7	20.9	20.2	0.36	0.555
	b*	30.0	29.9	29.9	29.8	30.0	0.26	0.962
Egg yolk color (Roche color fan)		5.6^c^	6.8^b^	7.7^a^	7.8^a^	7.7^a^	0.08	<0.001
Eggshell strength (kg/cm^2^)		4.69	4.76	4.84	4.65	4.95	0.126	0.465
Eggshell thickness (μm)		375	378	377	372	377	3.8	0.770
Haugh unit		90.9	90.0	91.9	91.4	91.2	1.26	0.860

Data are least squares means of 8 observations per treatment.

1) PC, recommended-energy diets with 2,900 kcal/kg AME_n_; NC, low-energy diets with 2,800 kcal/kg AME_n_; 0% MN, low-energy diets containing 5% PKM without MN supplementation; 0.05% MN, 0.05% MN supplementation in low-energy diets containing 5% PKM; 0.10% MN, 0.10% MN supplementation in low-energy diets containing 5% PKM.

a–c Means with different superscripts within a row differ (p<0.05).

SEM, standard error of the mean; CIE, Commission Internationale de l′Eclairage; L*, lightness; a*, redness; b*, yellowness.

**Table 4:** Effect of β-mannanase (MN) supplementation in low-energy diets containing palm kernel meal (PKM) on jejunal morphology in laying hens raised under heat stress conditions

Item	Dietary treatments1)					SEM	p-value

	PC	NC	PKM				

			0% MN	0.05% MN	0.10% MN		
VH (μm)	1,244^ab^	1,199^b^	1,215^b^	1,277^ab^	1,323^a^	29.8	0.042
CD (μm)	164^a^	162^ab^	158^c^	159^bc^	160^bc^	1.0	0.002
VW (μm)	157	157	155	157	157	1.7	0.810
VH:CD ratio	8.06	7.66	7.86	8.12	8.42	0.199	0.110

Data are least squares means of 8 observations per treatment.

1) PC, recommended-energy diets with 2,900 kcal/kg AME_n_; NC, low-energy diets with 2,800 kcal/kg AME_n_; 0% MN, low-energy diets containing 5% PKM without MN supplementation; 0.05% MN, 0.05% MN supplementation in low-energy diets containing 5% PKM; 0.10% MN, 0.10% MN supplementation in low-energy diets containing 5% PKM.

a–c Means with different superscripts within a row differ (p<0.05).

SEM, standard error of the mean; VH, villus height; CD, crypt depth; VW, villus width.

**Table 5:** Effect of β-mannanase (MN) supplementation in low-energy diets containing palm kernel meal (PKM) on liver visual characteristics in laying hens raised under heat stress conditions

Item		Dietary treatments1)					SEM	p-value

		PC	NC	PKM				

				0% MN	0.05% MN	0.10% MN		
Subjective color score		1.83	2.08	1.80	2.35	2.20	0.317	0.691
Hemorrhagic color score		1.05	1.23	1.53	2.00	1.60	0.267	0.135
Liver color (CIE Lab value)	L*	29.0	27.6	25.7	31.2	28.0	1.32	0.058
	a*	18.3^b^	17.5^b^	19.5^ab^	21.1^ab^	23.1^a^	1.32	0.037
	b*	10.3	9.8	9.9	13.9	13.0	1.41	0.134

Data are least squares means of 8 observations per treatment.

1) PC, recommended-energy diets with 2,900 kcal/kg AME_n_; NC, low-energy diets with 2,800 kcal/kg AME_n_; 0% MN, low-energy diets containing 5% PKM without MN supplementation; 0.05% MN, 0.05% MN supplementation in low-energy diets containing 5% PKM; 0.10% MN, 0.10% MN supplementation in low-energy diets containing 5% PKM.

a,b Means with different superscripts differ (p<0.05).

SEM, standard error of the mean; CIE, Commission Internationale de l’Eclairage; L*, lightness; a*, redness; b*, yellowness.

## Data Availability

Upon reasonable request, the datasets of this study can be available from the corresponding author.
